# IDH1 R132C and ERC2 L309I Mutations Contribute to the Development of Maffucci’s Syndrome

**DOI:** 10.3389/fendo.2021.763349

**Published:** 2021-11-01

**Authors:** Peng Cheng, Kun Chen, Shu Zhang, Ke-tao Mu, Shuang Liang, Ying Zhang

**Affiliations:** ^1^ Department of Orthopedics, Tongji Hospital of Tongji Medical College, Huazhong University of Science and Technology, Wuhan, China; ^2^ Department of Orthopedics, The First Affiliated Hospital of University of Science and Technology of China, Hefei, China; ^3^ The Center for Biomedical Research, Key Laboratory of Organ Transplantation, Ministry of Education and Chinese Academy of Medical Sciences, NHC Key Laboratory of Organ Transplantation, Huazhong University of Science and Technology, Wuhan, China; ^4^ Department of Radiology, Tongji Hospital of Tongji Medical College, Huazhong University of Science and Technology, Wuhan, China; ^5^ Department of Nephrology, Tongji Hospital of Tongji Medical College, Huazhong University of Science and Technology, Wuhan, China

**Keywords:** hemangiomas, Maffucci’s syndrome, ERC2 mutation, IDH1 mutation, multiple enchondromas

## Abstract

**Background:**

Maffucci’s syndrome is characterized by the coexistence of multiple enchondromas and soft-tissue hemangiomas. It has been clear that somatic mosaic isocitrate dehydrogenase type 1 (IDH1) or isocitrate dehydrogenase type 2 (IDH2) mutations are associated with Maffucci’s syndrome and Ollier disease, but the mechanisms underlying hemangiomas of the Maffucci’s syndrome is still obscure. This study aimed to determine the mechanism of hemangiomas in Maffucci’s syndrome.

**Methods:**

We received a 26-year-old female patient with typical Maffucci’s syndrome, and exome sequencing was conducted using DNA from her peripheral blood and enchondroma tissues. Somatic mutations were characterized by a comparative analysis of exome sequences and further confirmed by the sequencing of PCR products derived from original blood and tissue samples. The mutations of an additional 69 patients with Ollier disease were further tested. The functional impacts of these somatic mutations on Maffucci’s syndrome, especially the development of hemangiomas, were evaluated.

**Results:**

We reported a typical case of Maffucci’s syndrome, which was confirmed by both imaging findings and pathology. Through exome sequencing of this patient’s DNA samples, we identified an R132C mutation in the isocitrate dehydrogenase type 1 (IDH1) gene and an L309I mutation in the ELKS/RAB6-interacting/CAST family member 2 (ERC2) gene in this patient. Approximately 33.3% of the clones were positive for the IDH1 R132C mutation, and 19.0% of the clones were positive for the ECR2 L309I mutation. The IDH1 R132C mutation was detected in most of the patients with Ollier disease (51/69 patients), and the mean frequency of this mutation was 63.3% in total sequence readouts, but the ECR2 L309I mutation was absent in all of the patients with Ollier disease. *In vitro* experiments confirmed that the IDH1 R132C mutation promotes chondrocyte proliferation, and the ERC2 L309I mutation enhances angiogenesis.

**Conclusions:**

Our results suggest that while IDH1 is a known pathogenic gene in enchondromatosis, ERC2 is a novel gene identified in Maffucci’s syndrome. The somatic L309I mutation of ERC2 contributes to the pathogenesis of hypervascularization to facilitate the development of hemangiomas in Maffucci’s syndrome. The combination of the IDH1 R132C and ERC2 L309I mutations contributes to the development of Maffucci’s syndrome, and these results may enable further research on the pathogenesis of Maffucci’s syndrome.

## Introduction

Enchondromatosis is a rare, nonhereditary skeletal disorder with two common clinical subtypes, Maffucci’s syndrome and Ollier disease (1–3). Maffucci’s syndrome is characterized by the coexistence of multiple enchondromas and soft-tissue hemangiomas and has an incidence rate of 23% in cases of malignant tumors (4, 5), while Ollier disease shares the same characteristics of multiple enchondromas but does not involve hemangiomas. The estimated prevalence of Ollier disease is approximately 1/100,000, which is much more common than Maffucci’s syndrome (6–8).

It is clear that somatic mosaic isocitrate dehydrogenase type 1 (IDH1) and isocitrate dehydrogenase type 2 (IDH2) mutations are associated with Maffucci’s syndrome and Ollier disease (8, 9). Heterozygous mutations in IDH1 and IDH2 have also been detected in gliomas/glioblastomas (9, 10) and acute myeloid leukemia (AML) (11–13). IDH1 mutations usually occur at R132, and IDH2 mutations are generally found at R172, a residue analogous to R132 in IDH1 (14–16). However, an additional IDH2 mutation site, R140, has also been reported (17–19). The above somatic mutations render IDH1 or IDH2 unable to convert isocitrate to α-ketoglutarate but promote D-2-hydroxyglutarate accumulation, the levels of which strongly correlate with tumorigenesis (13, 20, 21). Other IDH1/IDH2 mutations have also been found, but the detailed functional relevance has not been described.

A monoallelic point mutation of IDH1 is believed to be strongly correlated with tumorigenesis, which explains the development of multiple enchondromas, but the mechanisms underlying hemangiomas in Maffucci’s syndrome have yet to be elucidated. We recently received a young female patient with Maffucci’s syndrome. To identify the possible pathogenic genes, we collected peripheral blood DNA and enchondroma DNA and conducted a comparative exome sequence analysis of the above DNA samples. We identified an R132C mutation in the IDH1 gene and an L309I mutation in the ELKS/RAB6-interacting/CAST family member 2 (ERC2) gene. We also obtained evidence suggesting that the IDH1 R132C mutation is likely the primary mutation responsible for the pathogenesis of multiple enchondromas, while the ERC2 L309I mutation may be the causative factor underlying hemangiomas by enhancing the intracellular calcium concentration in endothelial cells. Collectively, our data suggest that somatic mutations in these two genes synergistically contribute to the development of Maffucci’s syndrome.

## Materials and Methods

### Exome Sequencing and Somatic Mutation Analysis

Genomic DNA was isolated from the enchondroma tissues in right-hand finger bones and peripheral blood of the patient. Exome was captured and sequenced to 100× by BGI (Shenzhen Guangdong, China) using the Illumina HiSeq™ 2000 Sequencing Systems. The resulting sequences were first compared with published reference sequences (https://www.globus.org/) to exclude normal polymorphisms. Variants between enchondromas and peripheral blood DNA were analyzed by BGI to characterize somatic mutations with reads above 10% in the 100× readouts (22, 23). The mutations and frequencies were confirmed by randomly sequencing 21 PCR clones with a pMD^®^18-T vector (Takara Biotechnology, Dalian, China).

### Cell Culture

ATDC5 and HUVECs cells were purchased from the Chinese Academy of Cell Bank. ATDC5 cells were cultured with DMEM/F12 medium at 37°C with 5% CO_2_ in a humidified incubator. For chondrocyte induction, ATDC5 cells were cultured with CDM (chondrogenic differentiation medium) (Cyagen Biosciences Inc., China) at 37°C with 5% CO_2_ in a humidified incubator. HUVECs were cultured in 2% FBS EBM-2 endothelial cell basic medium at 37°C for 24 h under a 5% CO_2_ atmosphere.

### Lentivirus Vector Construction and Cell Infection

The coding sequence of the target gene (IDH1, IDH1 with R132C mutation, ERC2 and ERC2 with L309I mutation) was PCR amplified from the GV287 target gene using primers with AgeI/AgeI overhangs and cloned into pTZ58 (Fermentas, Vilnus, Lithuania). The AgeI/AgeI fragment was then subcloned into pUbi (AgeI/AgeI) and pEGFP-C1 (Clontech, Mountain View, CA) (AgeI/AgeI) to generate Ubi-GENE-3FLAG-SV40-EGFP encoding plasmids, respectively. To produce lentivirus, the pBABE-puro plasmids were coinfected along with the helper plasmids into 293T cells, and the medium was harvested 36 h and 72 h after infection (24). ATDC5 or HUVEC infection was performed by incubating the cells in virus-enriched medium for 12 h, which included 4 μg/ml polybrene. Transduced cells were identified for EGFP expression under a fluorescence microscope.

### Proliferation Assay

The cells (mouse chondrogenic cell line ATDC5 or HUVECs) were seeded in 96-well plates at a density of 2×10^4^ cells/well. For chondrocyte induction, 10 μg/ml bovine insulin (Wako Pure Chemical, Osaka, Japan) was added after 12 h of culture under a 5% CO_2_ atmosphere (21). After an additional 24 h of culture, 10 μl of WST-8 mixture (Dojindo, Shanghai, China) was added to each well and cultured for another 2 h. After washing, the cells were subjected to the measurement of absorbance under a microplate reader at a wavelength of 450 nm.

### Migration Assay

ATDC5 cells were seeded in a 24-well plate at a density of 8×10^4^ cells/well, and the cells were induced into chondrocytes as described above. A scratch was next created using a sterile yellow tip, the detached cells were removed, and the scratches were monitored for 48 h under culture conditions without any bovine insulin. Each set of experiments was performed in triplicate, and photographs were taken at the indicated time points.

### Transwell Invasion Assay

ATDC5 cells or HUVECs were placed on the upper layer of culture medium inserted with a permeable membrane, and BD Matrigel (BD Biosciences, San Jose, CA) was placed below the cell permeable membrane. After 12 h of incubation, the ATDC5 cells that migrated through the membrane were stained with crystal violet solution and then counted under a light microscope, while the migrated HUVECs were analyzed under a fluorescence microscope.

### Cell Cycle Analysis

The transduced ATDC5 cells were first induced to chondrocytes as described earlier and then synchronized to the G_0_/G_1_ phase by 24 h of serum starvation, and 10% FBS was added to the cultures. After another 24 h of culture, the cells were harvested for cell cycle analysis. Briefly, after washing, the cells were stained with propidium iodide, and the cell cycle distribution for each culture was analyzed by flow cytometry.

### Tube Formation Assay

To examine tube formation, growth factor-reduced Matrigel (BD Bioscience, San Jose, CA) was placed in 96-well tissue culture plates (100 μL/well) and allowed to form a gel at 37°C for at least 30 min. HUVECs (2 x 10^4^ cells) after 24 h of transduction were added into each well and incubated in 2% FBS EBM-2 endothelial cell basic medium at 37°C for 24 h under a 5% CO_2_ atmosphere. Endothelial tubes were then examined under a fluorescence microscope by inspecting the overall tube length and branch points.

### Intracellular Free Calcium Assay

The transduced HUVECs were seeded in a 24-well plate at a density of 2×10^4^ cells/well. After 12 h of culture, cell-permeant acetoxymethyl (AM) esters of X-Rhod-1 were loaded into the cultures. After another 4 h of culture, the cells were subjected to analysis of intracellular calcium concentration under an Olympus IX73 fluorescence microscope at 550 nm excitation and 600 nm emission. Images were taken at 100X magnification.

### Statistical Analysis

For pairwise comparisons, the data were analyzed using Student’s t-test. A comparison between multiple experimental groups was accomplished by one-way ANOVA using SPS 11.5 for windows. All experiments were conducted with at least 3 independent replications. All data are presented as the mean ± SEM. In both cases, p < 0.05 was considered to be statistically significant.

## Results

### Clinical Report

**Figure 1:**
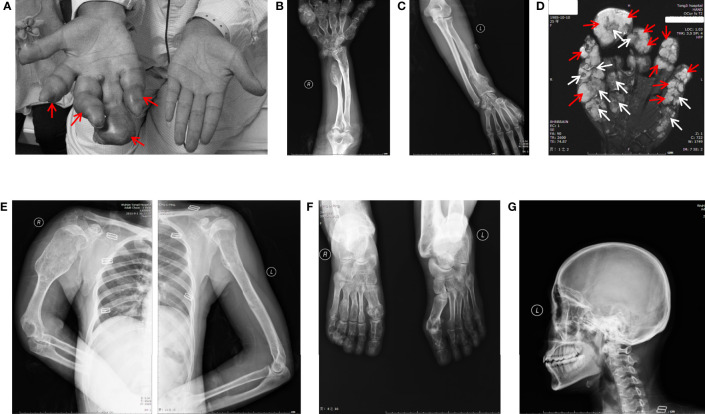
General view and radiographic imaging of the lesion. **(A)** The right hand of the patient with Maffucci’s syndrome showed deformities due to multiple enchondromas and a superficial hemangioma. The red arrows show hemangioma as a soft spherical bulge in light blue. **(B)** X-ray images of the right ulnar and radial bones and multiple metacarpophalangeal bones of the right hand show multiple reduced bone densities and partial expansive bone destruction **(C)**. The X-ray shows a relatively normal bone mass in the left arm. **(D)** MR results of the patient’s right hand showed the phalanges are circular or oval, multilocular, well-defined, iso-intensity and hyperintensity on T2WI (white arrows) and the soft tissue around the phalanx presents dilatative, well-bounded hyperintensity on T2WI (red arrows). **(E)** X-ray images of the patient’s upper body and enlarged right arm are shown in the film. **(F)** X-ray images of the patient’s feet have the same radiographic appearance as those of the upper limbs. **(G)** X-ray image of cranial and maxillofacial bones showed no apparent abnormalities.

The patient here we report is a 26-year-old young woman who had typical multiple enchondromas but had no family members with disease history. Physical exams revealed that her 3-year-old daughter was also normal. The patient had abnormal protrusion in her right-hand fingers when she was 1 year old. Unfortunately, no medical examination was conducted at that time because of her family’s financial problems. The patient presented when she was 26 years old; by then, all of her limbs could still move but were affected by deformities. The right hand had dangerous tumor-like deformities, fingers in this hand displayed deformity shape of the visible nodular and soft spherical bulge in light blue (**Figure 1A**), and the right upper arm was shortening (**Figure 1B**), but her left hand showed healthy appearance (**Figure 1C**). The right knee showed strong varus, and the left knee displayed valgus; both feet and ankles displayed deformities (data not shown). Palpable subcutaneous nodules could be found around the bones of these deformity areas, but no tenderness was characterized.

Ultrasound examinations revealed that her liver, gallbladder, spleen, pancreas, pancreatic duct, kidneys, and bladder were healthy. MR examinations showed that the phalanges of the fingers are circular or ovoid, with multi-locular, well-defined chondromatoid lesions and the soft tissue around the phalanx of the finger presents dilatative, well-bounded hyperintensity on T2WI.(**Figure 1D**). X-ray examinations demonstrated that the bones of the right palm, fingers of the right hand, right ulna, both radius (**Figure 1B**), humerus, scapulas, head of the right seventh rib (**Figure 1E**) and metatarsals of both feet (**Figure 1F**) were characterized by irregular swelling and morphological abnormalities. Cortical bones of these parts were markedly thinned, and multiple cystic-like lucent and dotted calcification areas can be noted around these parts. Bones in the cranial and maxillofacial regions, however, had no apparent abnormalities (**Figure 1G**). These asymmetrically distributed enchondroma bone destruction and surrounding soft tissue hemangioma malformations are typical imaging features of Maffucci’s syndrome.

**Figure 2:**
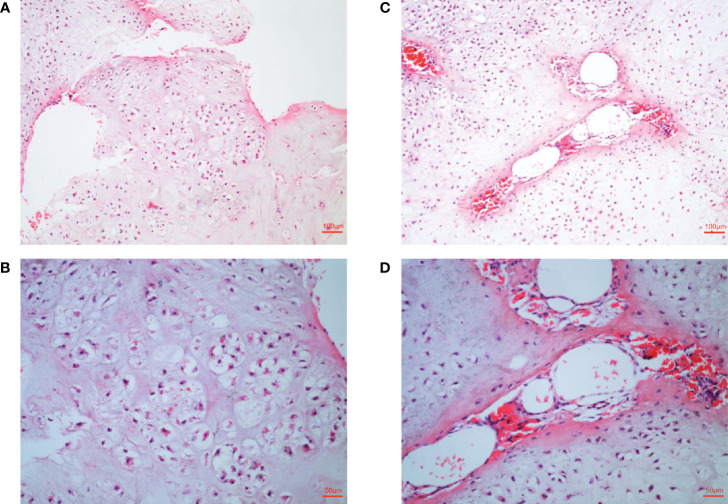
Histological examination of lesions. **(A, B)** Histological examination manifesting typical enchondroma in the patient’s right-hand finger bones. The specific manifestation was microscopic appearance of lobular hyaline cartilage with uniform chondrocytes, heaps of chondrocytes, uniform nuclear size and not deep staining **(C, D)**. Samples were collected from patient’s right-hand finger bones displaying cavernous hemangioma in the enchondroma tissues. Microscopically, a large number of neoplastic vascular tissues can be seen in the middle of cartilage tissue. The blood vessels are thin-walled and dilated and filled with red blood cells.

These lesions seriously affected the function of the patient’s hands, and the patient was very concerned that these lesions may be malignant. In order to further clarify the disease and nature, biopsy was necessary. Histological analysis indicated typical enchondroma changes in sections originating from right finger bones (**Figures 2A, B**), and cavernous hemangioma changes were noted in the enchondroma tissues from the same finger (**Figures 2C, D**).

According to these typical clinical manifestations, radiological data, and histological analysis by the authoritative pathologist, the diagnosis of Maffucci’s syndrome was clear.

### Exome Sequencing and Characterization of Somatic Mutations in the IDH1 and ERC2 Genes in Maffucci’s Syndrome

Exome sequencing was next conducted using the patient’s DNA samples originating from her peripheral blood and enchondroma tissues with 100x coverage. Comparative analysis of her enchondroma exome sequences with the sequences of 1000 standard human specimens in the public database Globus (https://www.globus.org/) revealed more than 65,000 variations, more than 99% of which are likely DNA polymorphisms between individuals. We thus first excluded those common polymorphisms identified through the public database and then aligned her enchondroma exome sequences with her peripheral blood exome sequences, *via* which we identified 90 mutations that cause amino acid changes. In general, somatic mutations in the enchondroma tissues are likely mosaic because of normal cell contamination or very low frequencies (only a proportion of tumor cells carry the same somatic mutation). By exclusion of those possibilities, the differences were limited to two mutations: the C394T mutation of IDH1 (NM_005896) in exon 4, which causes arginine to cysteine at position 132 (R132C), and the frequency of this mutation is 38.7% in total sequence readouts; and the C925A mutation of ERC2 (NM_015576) in exon 3, which mutates leucine to isoleucine at position 309 (L309I), and the ratio of this mutation is 23.8% in total sequence readouts.

**Figure 3:**
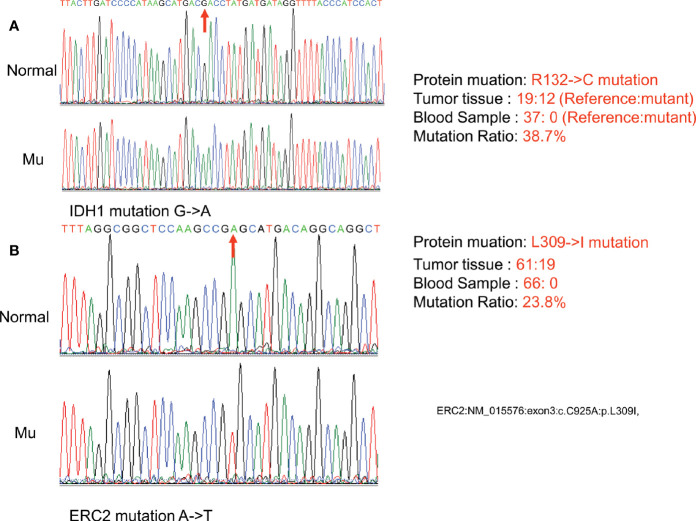
Confirmation of somatic nonsynonymous mutation of IDH1 (c. C394T) **(A)** and ERC2 (c. C925A) **(B)** by sequencing PCR clones. The fragment flanking the mutated nucleotide was amplified by PCR and then cloned into a pMD18-T vector. Twenty-one clones were randomly sequenced to confirm the mutations and their frequency. (Normal:Normal; Mu:Mutation).

To confirm the above sequencing data, we next PCR amplified the two regions (IDH1-c. C394T and ECR2-c. C925A) from her peripheral blood DNA and enchondroma DNA, respectively. The resulting PCR products were cloned into a TA vector, followed by sequencing analysis of 21 randomly selected clones. Indeed, these mutations were absent in the blood DNA, while approximately 33.3% of the clones were positive for the IDH1 R132C mutation (**Figure 3A**), and 19.0% of the clones were positive for the ECR2 L309I mutation (**Figure 3B**). To exclude the possibility that those mutations are also present in healthy individuals, we genotyped 500 healthy individuals, and none of the subjects detected those two mutations.

### IDH1 R132C Mutation Is in Both Subtypes of Enchondromatosis, While ERC2 L309I Mutation May Only Be in Maffucci’s Syndrome

**Figure 4:**
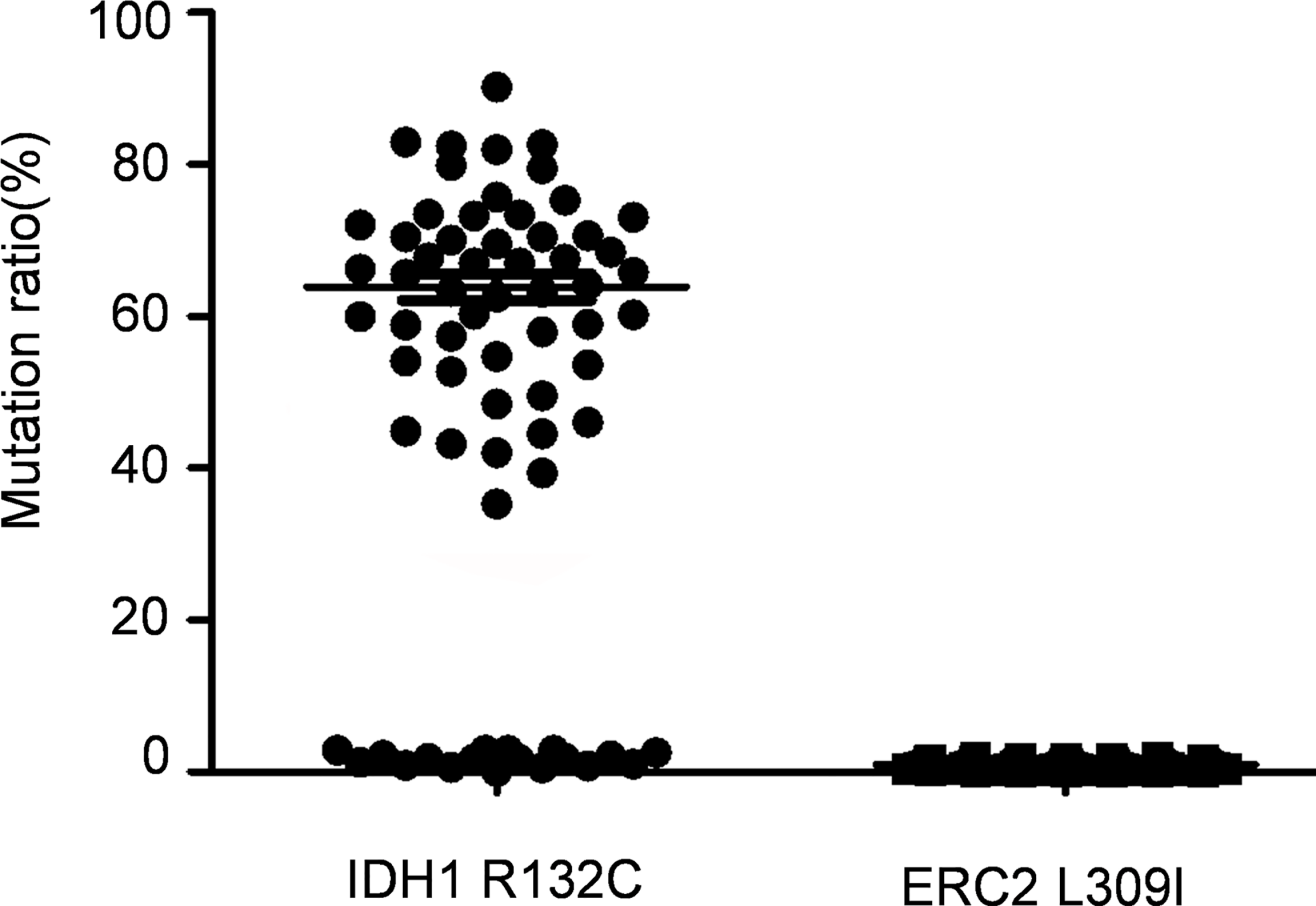
The IDH1 R132C mutation is in Ollier disease, while the ERC2 L309I mutation is absent. The IDH1 R132C mutation was detected in most of the patients with Ollier disease (51/69 patients), and the mean frequency of this mutation was 63.3% in total sequence readouts, but the ECR2 L309I mutation was absent in all patients with Ollier disease examined.

The foregoing case of Maffucci’s syndrome demonstrates that there may be two gene mutations of IDH1 and ERC2 in Maffucci’s syndrome. To demonstrate additional evidence of these two mutations in disease pathobiology, we genotyped 69 patients with Ollier disease using DNAs extracted from enchondroma tissues after dissection of embedded paraffin blocks. Excitingly, the IDH1 R132C mutation was detected in most of the patients with Ollier disease (51/69 patients), and the mean frequency of this mutation was 63.3% in total sequence readouts, but the ECR2 L309I mutation was absent in all of the patients with Ollier disease (**Figure 4**). Unfortunately, we were unable to identify additional patients with Maffucci’s syndrome for the analysis of the ECR2 L309I mutation.

### The IDH1 R132C Mutation Promotes Chondrocyte Proliferation, and the ERC2 L309I Mutation Enhances Angiogenesis

**Figure 5:**
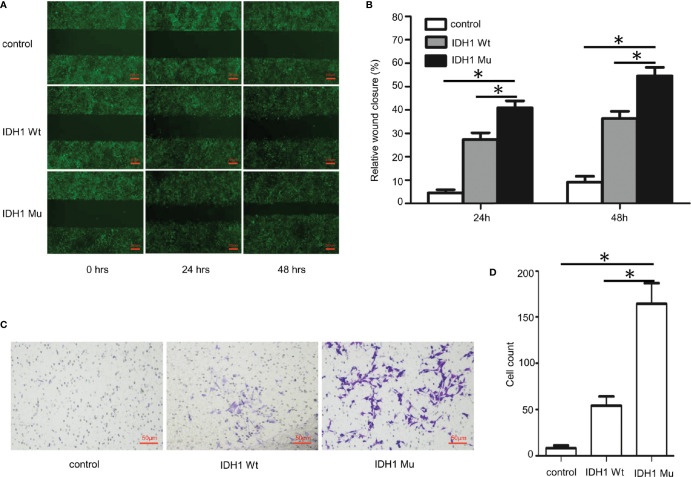
Impact of the IDH1 R132C mutation on chondrocyte migration. The mouse chondrogenic ATDC5 cells were transduced with IDH1 wt and IDH1 mu (R132C) lentiviruses and then induced into chondrocytes as described. ATDC5 cells transduced with empty lentiviruses served as a control. **(A)** Representative images of each group from typical scratch migration assays. **(B)** A bar graphic figure shows the results of 5 independent scratch migration assays conducted. **(C)** Representative images of each group from transwell migration assays. **(D)** A bar graph shows data collected from 3 independent transwell migration assays. (*P<0.05).

**Figure 6:**
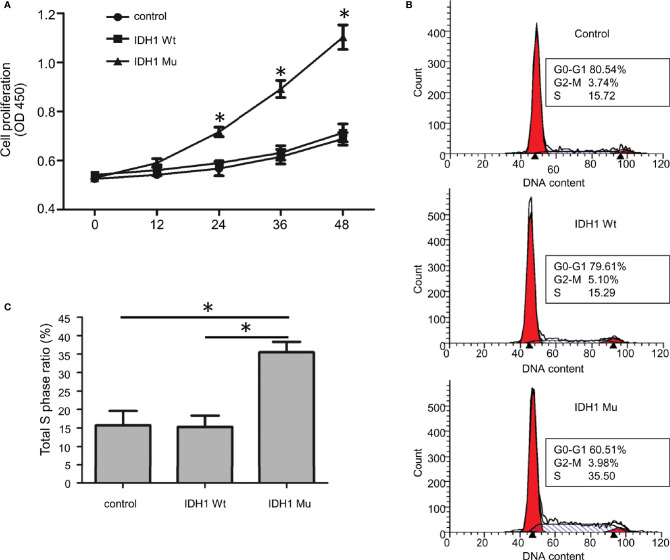
The effect of the IDH1 R132C mutation on chondrocyte growth. **(A)** Proliferation results of chondrocytes after staining with WST-8 (n = 3). **(B)** Representative results of each group for cell cycle analysis. **(C)** A bar graphic figure shows the results collected from 3 independent replications. The IDH1 mu transduced cells manifested a significantly higher capacity for G1-S phase transition. (*P<0.05).

Given that the ECR2 L309I mutation is absent in patients with Ollier disease (with multiple chondromas only), while Maffucci’s syndrome is characterized by the coexistence of multiple chondromas and hemangiomas, we thus hypothesized that the IDH1 R132C mutation causes multiple chondromas, while the ERC2 L309I mutation is responsible for the development of hemangiomas. To test this hypothesis, we conducted studies in chondrocytes, in which we induced a mouse chondrogenic cell line, ATDC5, into chondrocytes after transduction with lentiviruses expressing wild-type IDH1 (IDH1-wt) or the R132C mutant (IDH1-mu) (22, 23). As expected, chondrocytes transduced with the IDH1-mu viruses exhibited significantly higher migration capacity (**Figure 5**) and proliferation capability (**Figure 6A**). Cell cycle analysis revealed that the R132C mutation significantly promoted the G1-S phase transition (**Figures 6B, C**).

**Figure 7:**
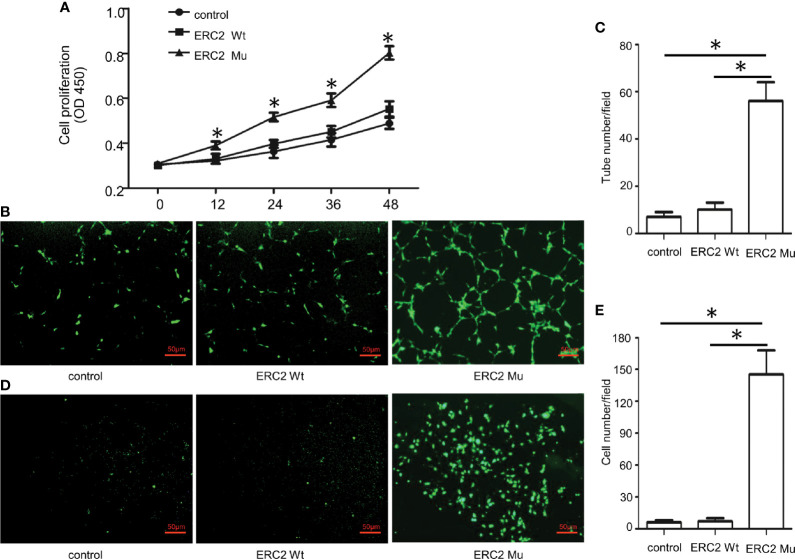
The effects of the ERC2 L309I mutation on endothelial angiogenesis. HUVECs were transfected with ERC2 wt and ERC2 mu (L309I) lentiviruses and then subjected to angiogenesis analysis. **(A)** Proliferation results by analysis of WST-8 fluorescence (n = 3). **(B)** Representative images for analysis of tubular formation. **(C)** Quantitative results for capillary-like tube formation (n = 3). **(D)** Representative images for transwell migration assays. **(E)** A bar graph displaying the data from 3 independent transwell migration assays. (*P<0.05).

To demonstrate the impact of the ERC2 L309I mutation on the development of hemangiomas, we checked its role in angiogenesis, as hemangiomas are characterized by excessive vessel formation. For this purpose, we transduced HUVECs with lentiviruses expressing wild-type ERC2 (ERC2-wt) and the L309I mutant (ERC2-mu). Interestingly, the transduction of ERC2-mu viruses potently enhanced the capacity of HUVECs for angiogenesis, as evidenced by the higher capability for proliferation (**Figure 7A**), tubular formation (**Figures 7B, C**) and migration (**Figures 7D, E**). Collectively, these data suggest that the IDH1 R132C mutation combines with the ERC2 L309I mutation to cause the development of Maffucci’s syndrome.

### The ERC2 L309I Mutation Increases the Concentration of Intracellular Calcium

**Figure 8:**
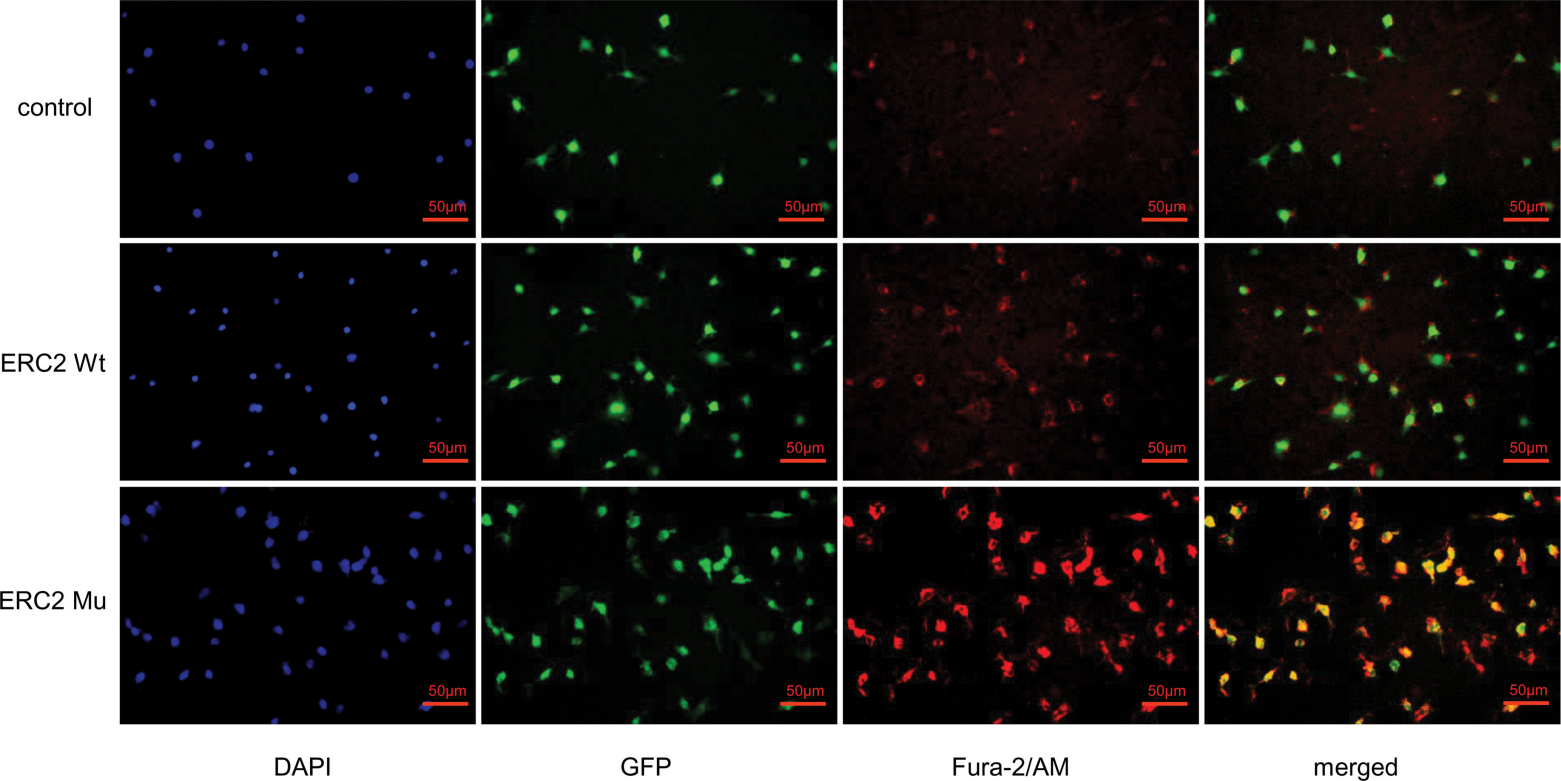
ERC2 L309I mutation enhances endothelial intracellular calcium concentration. HUVECs were transduced with ERC2 wt and ERC2 mu (L309I) lentiviruses. HUVECs transduced with empty lentiviruses served as a control. Fura-2/AM was next applied to the cultures, and the intracellular free calcium concentration was then assessed for red fluorescence after washing under a fluorescence microscope.

Given that CAST/ERC2 has been noted to modulate neurotransmitter release in nerve terminals by regulating intracellular Ca^2+^ concentrations (24), we then examined the effect of the L309I mutation of ERC2 on the intracellular calcium concentration in HUVECs by staining with Fura-2/AM (Invitrogen, OR, USA), a dye used to measure intracellular free calcium. HUVECs transduced with ERC2-mu displayed significantly higher intracellular free calcium concentrations than cells transduced with ERC2-wt (**Figure 8**).

## Discussion

Maffucci’s syndrome is characterized by the coexistence of multiple enchondromas and soft-tissue hemangiomas (4, 5). Multiple enchondromas are characterized by irregular distribution of multiple benign cartilaginous lesions within the bones (7, 25). The phalanges, femur, and tibia are most commonly affected, with a tendency towards unilateral involvement (8, 26). As this patient showed, there is typical deformity in a limb or multiple painless bony lesions in the right hands, and the deformity is asymmetrically distributed. Radiographs typically show multiple well-defined lytic lesions within the medullary canal with a thin overlying cortex. Calcification can also be seen within the lesion, and the bone is enlarged, shortened, and deformed (4). Histological analysis indicated typical enchondroma changes, and cavernous hemangioma changes were noted in sections derived from the right palm. Therefore, the patient’s diagnosis of Maffucci’s syndrome was correct.

Maffucci’s syndrome has a 23% incidence of a malignant tumor (8). It has been reported that somatic mosaic isocitrate dehydrogenase type 1 (IDH1) or isocitrate dehydrogenase type 2 (IDH2) mutations are associated with Maffucci’s syndrome (10, 27). R132 of IDH1 is a hotspot somatic mutation site that has been reported to be the leading cause of chondrosarcoma/chondromas (10, 28), gliomas/glioblastomas (29) and some type of AML (30). Indeed, epigenetic studies suggest that this mutation was sufficient to establish the glioma hypermethylation phenotype in a cell model (29). In this patient, R132 was mutated to cysteine (R132C), and we confirmed that it was the primary mutation responsible for the development of multiple enchondromas.

In contrast, the ERC2 (L309I) mutation was a novel discovery, and its impact on the pathogenesis of Maffucci’s syndrome is entirely unknown. The function of ERC2 is more reported in the release of neurotransmitters. In nerve terminals, CAST/ERC2 forms a protein complex with other active zone proteins and is thought to play an organizational and functional role in neurotransmitter release (31–33). Studies have also reported that genetic aberrations of ERC2 accelerate tumor formation in the body, such as kidney cancer and pancreatic cancer (34). Our results demonstrate the impact of the ERC2 L309I mutation on the development of hemangiomas, which may be an essential factor in the transformation of enchondromatosis into Maffucci’s syndrome.

After further study, we found higher intracellular free calcium concentrations in HUVECs transduced with ERC2-mu. This result indicates that the L309I mutation rendered HUVECs with higher potency to regulate intracellular calcium influx (35). Since intracellular Ca^2+^ is known to be a second messenger for signal transduction closely related to cell proliferation, migration, apoptosis, and survival (36–39), our data suggest that the ECR2 L309I mutation probably contributes to the development of hemangiomas by enhancing intracellular calcium concentrations in endothelial cells.

In summary, by a comparative analysis of exome sequences between peripheral blood DNA and enchondroma DNA in a patient with Maffucci’s syndrome, we identified an R132C mutation in the IDH1 gene and an L309I mutation in the ERC2 gene. Initial functional studies suggest that the IDH1 R132C mutation is likely the primary mutation responsible for the development of enchondromas, while the ERC2 L309I mutation is probably a causative mutation underlying the pathogenesis of hemangiomas. Therefore, our results suggest that the R132C mutation in IDH1 and the L309I mutation in ERC2 are probably the causative factors contributing to the development of Maffucci’s syndrome. These data may promote further research on the pathogenesis of Maffucci’s syndrome.

## Data Availability Statement

The original contributions presented in the study are included in the article/supplementary material. Further inquiries can be directed to the corresponding author.

## Ethics Statement

The studies involving human participants were reviewed and approved by Ethics Committee of Tongji Hospital Affiliated to Tongji Medical College (TJMC). The patients/participants provided their written informed consent to participate in this study. Written informed consent was obtained from the individual(s) for the publication of any potentially identifiable images or data included in this article.

## Author Contributions

YZ designed experiments and edited the manuscript. PC and KC conducted most of the experiments, analyzed the data and wrote the draft. YZ collected the biopsy samples and did pathological analysis. SZ and SL conducted mutation analysis. PC followed up the patient and obtained the clinical data. K-tM collected imaging data and provided professional advice. All authors contributed to the article and approved the submitted version.

## Funding

This work was supported by the Hubei Province health and family planning scientific research project (Grant number WJ2019Q028) and Natural Science Foundation of Hubei Province of China (Grant No. 2020CFB216).

## Conflict of Interest

The authors declare that the research was conducted in the absence of any commercial or financial relationships that could be construed as a potential conflict of interest.

## Publisher’s Note

All claims expressed in this article are solely those of the authors and do not necessarily represent those of their affiliated organizations, or those of the publisher, the editors and the reviewers. Any product that may be evaluated in this article, or claim that may be made by its manufacturer, is not guaranteed or endorsed by the publisher.
